# Validation of an UHPLC-MS/MS Method for Screening of Antimicrobial Residues in Eggs and Their Application to Analyses of Eggs from Laying Hens Subjected to Pharmacological Treatment

**DOI:** 10.1155/2017/3259073

**Published:** 2017-10-23

**Authors:** Letícia Gomes Magnago Caldeira, Flávio Alves Santos, Andréa Melo Garcia de Oliveira, Josefa Abucater Lima, Leonardo Francisco de Souza, Guilherme Resende da Silva, Débora Cristina Sampaio de Assis, Silvana de Vasconcelos Cançado

**Affiliations:** ^1^Escola de Veterinária, Universidade Federal de Minas Gerais (UFMG), Av. Antônio Carlos 6627, 30.123-970 Belo Horizonte, MG, Brazil; ^2^Laboratório Nacional Agropecuário (Lanagro-MG), Av. Rômulo Joviano s/n, 33.600-000 Pedro Leopoldo, MG, Brazil

## Abstract

A multiresidue method by UHPLC/MS-MS was optimized and validated for the screening and semiquantitative detection of antimicrobials residues from tetracyclines, aminoglycosides, quinolones, lincosamides, *β*-lactams, sulfonamides, and macrolides families in eggs. A qualitative approach was used to ensure adequate sensitivity to detect residues at the level of interest, defined as maximum residue limit (MRL), or less. The applicability of the methods was assessed by analyzing egg samples from hens that had been subjected to pharmacological treatment with neomycin, enrofloxacin, lincomycin, oxytetracycline, and doxycycline during five days and after discontinuation of medication (10 days). The method was adequate for screening all studied analytes in eggs, since the performance parameters ensured a false-compliant rate below or equal to 5%, except for flumequine. In the analyses of eggs from laying hens subjected to pharmacological treatment, all antimicrobial residues were detected throughout the experimental period, even after discontinuation of medication, except for neomycin, demonstrating the applicability of the method for analyses of antimicrobial residues in eggs.

## 1. Introduction

Antimicrobials have been widely used in veterinary medicine as therapeutic or prophylactic agents. However, the use of such medications may result in the presence of their residues in eggs, which may cause allergic reactions or toxicity or lead to the selection of antibiotic-resistant microorganisms in humans [1]. Because of the ovarian follicle development and of preovulatory hierarchy, many weeks may be required following treatment or exposure before eggs are free of some drug residues [2].

To ensure food safety, study and determination of the appropriate withdrawal period of antimicrobials used in the treatment of the hens as well as an efficient residue control in food products from animal origin are essential. Maximum residue limits (MRL) for veterinary drug residues in foods of animal origin were established by European Regulation number 37/2010 and the Codex Alimentarius Commission, based on the acceptable daily intake (ADI) of each drug and considering maximum food intake [3–5]. Nevertheless, to ensure compliance with these regulations, sensitive and specific analytical methods, capable of monitoring quickly and efficiently the presence and the level of these residues, are necessary [6, 7].

The analytical methods for determination of veterinary drugs residues in foods may be classified as screening or as quantitative and confirmatory methods. Microbiological inhibitory plate test methods may be used for the screening of antimicrobial residues. However, due to the presence of inhibitory substances, especially in the albumen, which serve as natural defense against microbial contamination and proliferation, the eggs are not considered a common product to be tested by microbiological inhibitory tests. Furthermore, these methods are not sensitive enough and not really specific; therefore a further postscreening step is necessary in order to determine the identity of the previously detected inhibitory substance [8]. Screening methods that employ ultra-performance liquid chromatography coupled to tandem mass spectrometry (UHPLC-MS/MS) techniques may be used as a semiquantitative postscreening tool, providing the unambiguous identification of the analytes of interest and the information about the compliance of the analyzed samples with the MRL established by regulatory agencies or a specific level of interest [9].

Despite the number of papers available in the literature about the development and validation of analytical methods for study of veterinary drug residues in foods of animal origin, there are few articles about the determination of antimicrobial residues in eggs by UHPLC-MS/MS, due to the complexity of the egg matrix. The papers available in the literature do not evaluate some classes of substances, such as the aminoglycosides [7, 10–14]. Furthermore, some of the developed methodologies are not validated [7, 12] or have recoveries for some analytes outside of the recommended limits for validation [11]. According to the EURACHEM [15], when the matrix used in the validated methodology changes, a new validation should be performed, since a standard method can not be used outside the scope for which it was designed. Moreover, most published studies that have evaluated the withdrawal period of antimicrobials in laying hens used microbiological assays [16–18] or other techniques, such as high-performance liquid chromatography (HPLC) or HPLC-mass spectrometry [19–23]. Thus, there is a lack of papers in the scientific literature on the elimination of these residues in eggs by UHPLC-MS/MS, as well as the evaluation of the compliance or not of the egg samples from laying hens subjected to a pharmacological treatment in relation to the MRL established by regulatory agencies.

Thus, the purpose of this work was to optimize and validate a qualitative and confirmatory method for screening of antimicrobials from tetracyclines, aminoglycosides, quinolones, lincosamides, *β*-lactams, sulfonamides, and macrolides families in eggs by UHPLC-MS/MS and evaluate its application as a semiquantitative screening method for detection of antimicrobial residues in eggs from hens that had been subjected to a pharmacological treatment.

## 2. Materials and Methods

### 2.1. Chemicals and Reagents

The analytical standards used for the tetracyclines (chlortetracycline, doxycycline, oxytetracycline, and tetracycline) and aminoglycosides (amikacin, apramycin, dihydrostreptomycin, gentamicin, hygromycin, kanamycin, neomycin, spectinomycin, streptomycin, and tobramycin) families were purchased from Sigma Chemical Co. (St. Louis, MO, USA). For the quinolones family, the standards of enrofloxacin, flumequine, norfloxacin, oxolinic acid, and sarafloxacin were obtained from Sigma Chemical Co., whereas the ciprofloxacin and the nalidixic acid were purchased from the Acros Organics (Geel, Belgium) and from the CDN Isotopes (Quebec, Canada), respectively. The standard of lincomycin, from the lincosamides family, as well as the standards from the macrolides (clindamycin, erythromycin, spiramycin, tilmicosin, and tylosin) and *β*-lactams (cefazolin, cloxacillin, dicloxacillin, oxacillin, nafcillin, penicillin G, and penicillin V) families were purchased from Sigma Chemical Co. For the sulfonamides family, the standards of sulfachloropyridazine, sulfadiazine, sulfadimethoxine, sulfadoxine, sulfamerazine, sulfamethazine, sulfamethoxazole, sulfamethoxypyridazine, sulfaquinoxaline, sulfathiazole, and sulfisoxazole were obtained from Sigma Chemical Co., whereas the standards of sulfadiazine and sulfathiazole were obtained from Dr. Ehrenstorfer Standards (Augsburg, Germany). All reagents used were of pro analysis (p.a.) grade, except the solvents used in UHPLC, which were of HPLC grade.

### 2.2. Standard Solutions

The purchased antimicrobial standards were used for the preparation of individual standard stock solutions in methanol or water, depending on the solubility of each antimicrobial at concentrations of 200 *μ*g mL^−1^ (tetracyclines, *β*-lactams, and aminoglycosides), 1000 *μ*g mL^−1^ (quinolones and sulfonamides), and 100 *μ*g mL^−1^ (lincomycin and macrolides).

Two working mixed standard solutions were prepared. The first, used in the extraction procedure by trichloroacetic acid (TCA), was diluted with ultrapure water (0.1 *μ*g mL^−1^ for quinolones, 4 *μ*g mL^−1^ for tetracyclines, 0.5 *μ*g mL^−1^ for lincosamides, and 5 *μ*g mL^−1^ for the aminoglycosides) and remained stable for 30 days when stored at −20°C. The second working mixed standard solution, used in the extraction procedure by acetonitrile, was also prepared in ultrapure water (0.1 *μ*g mL^−1^ for sulfonamides, 0.5 *μ*g mL^−1^ for *β*-lactams, and 1.5 *μ*g mL^−1^ for the macrolides, except for tylosin that was prepared at a concentration of 2.0 *μ*g mL^−1^) and remained stable for one week when stored at −20°C. All standard solutions were stored protected from light in amber bottles.

### 2.3. Sample Preparation

The internal content of the eggs (albumen and yolk) was homogenized using Ultra Turrax (IKA®, Wilmington, NC, USA) and then 2.0 g of samples was weighed in 50 mL polypropylene centrifuge tubes. Then, two extractions procedures, adapted from Gaugain-Juhel et al. [8], followed by two acquisition methods were employed to allow the screening of all 45 studied antimicrobials.

### 2.4. Extraction of Tetracyclines, Aminoglycosides, Quinolones, and Lincosamides

The analytes were extracted from 2 g egg samples with 8 mL of 5% TCA solution. The tubes were mixed for 10 minutes in an orbital shaker. Then, 1.5 mL of the obtained extract was transferred to centrifuge microtubes and centrifuged (14.462 ×g), at 4°C, for 12 minutes in a refrigerated centrifuge (SIGMA 3-30KS®, ATR, Laurel, MD, USA). After centrifugation, the supernatant was filtered through a filter unit with polytetrafluoroethylene (PTFE) membrane (pore size of 0.22 *μ*m, diameter 13 mm, FilterPro®) and the filtrate was transferred to a vial for injection. The extraction procedure was adapted from the method described by Gaugain-Juhel et al. [8].

### 2.5. Extraction of Macrolides, *β*-Lactams, and Sulfonamides

The samples were added to 8 mL of acetonitrile, stirred for 10 min, and then centrifuged at 3000 ×g at 4°C, for 10 minutes in a refrigerated centrifuge (SIGMA 3-30KS, ATR). The supernatant (6 mL) was evaporated under nitrogen flow at 40 ± 3°C and the obtained extract was dissolved in 0.6 mL of 0.2 M ammonium acetate solution and filtered through a filter unit with PTFE membrane (pore size of 0.22 *μ*m, diameter 13 mm, FilterPro) and the filtrate was transferred to a vial insert for injection. The method of extraction was adapted from Gaugain-Juhel et al. [8].

### 2.6. Instrumentation

Chromatographic separation was performed in an UHPLC system (Prominence Shimadzu), using an Agilent Zorbax Eclipse XDB C18 3.5 *μ*m × 4.6 × 30 mm, with a vanguard column. The gradient mixing 0.2% heptafluorobutyric acid (HFBA) (mobile phase A) and acetonitrile (mobile phase B) was used for the separation of the tetracyclines, aminoglycosides, quinolones, and lincosamides. Initial conditions were set at 10% B with a linear gradient from 10% B to 50% from 0.01 to 7 minutes, and then 50% B was held for 4 min with an immediate return to 10% B at 12 min. The total run time for each injection was 13 min. The gradient optimized for the separation of macrolides, *β*-lactams, and sulfonamides, mixing 0.1% HFBA (mobile phase A) and acetonitrile (mobile phase B), started with 10% B. It was then increased linearly to 30% of B over 4 min and then stopped for 1 min at 30% and again raised linearly to 70% of B over 3 min and stopped for 3 min at 70%. The initial composition was then recovered over a 5-min delay. The total run time was 16 min. The flow rate was set at 0.6 mL min^−1^ and the partial loop with needle overfills injection volume was 20 *μ*L in both cases.

For the detection and identification of the targeted analytes, a 4000 QTRAP® triple quadrupole mass detector was used (AB Sciex, Darmstadt, Germany), set in positive ESI mode. The capillary voltage was set at 5.5 kV and the temperature of the source at 650°C. Declustering potential (DP) and the collision energy (CE) were optimized for each analyte by infusing solutions of the antimicrobial standards prepared in the mobile phase, in order to improve the signal intensity. Nitrogen was used as collision gas at 8.0 psi and curtain gas at 20.0 psi. Two MRM transitions were established and monitored for each analyte. The major transition (1st transition) was used for analyte identification and the minor transition (2nd transition) for their confirmation. The presence of two MRM transitions with a signal-to-noise ratio (S/N) > 3 in combination with the expected retention time guarantees the univocal identification of the analyte.

### 2.7. Validation Procedure

The UHPLC-MS/MS method was validated by referencing the validation procedure to monitor antimicrobial residues in milk described by Gaugain-Juhel et al. [8]. According to these authors, the proposed scheme of validation, applied to an UHPLC-MS/MS postscreening method, is more suitable than the classical approach of validation usually applied to quantitative methods that check parameters, such as trueness, precision, and linearity. The evaluation of many samples at the level of interest, assessing statistically the capacity of detection of the method, was considered more relevant, because the aim of a validation is to prove the suitability of the method in achieving the goal for which it is developed. Generally, the level of interest corresponds to the MRL level and the samples are spiked at this concentration for the validation. However, for some compounds, for which MRL is established for the parent drug plus its metabolite or for different compounds (e.g., tetracyclines), a level of interest lower than the MRL, such as 0.75 MRL, may be chosen [3, 5, 8]. Thus, the validation procedure was performed at different levels of interest, according to the MRL established for each drug (Table 1).

The parameters evaluated in this work were selectivity [6], *T*-value, cut-off factor (Fc), limit of detection (LOD), detection capability (CC*β*), and sensitivity [8, 24].

The selectivity of the method was evaluated by analyses of 20 blank samples, from different batches, and from laying hens that were not treated with antimicrobials, to check the presence of any interferences (signals, peaks, and ion traces) in the region of interest, which elute at the same retention time as the target analytes [6].

The calculation of the *T*-value for each compound of interest is a first step in assessing the capacity of detection. *T*-value is a “threshold” value corresponding to the minimum analytical response above which the sample will be truly considered as positive. This parameter was determined by analyses of 20 blank samples from different origins and calculated using the equation: *T*-value = *B* + 1.64 × SD_*B*_, which considers the mean value of the noise “*B*” and the standard deviation of 20 recorded noises “SD_B_” [8].

The LOD was calculated using the equation: LOD = 3*B* × *C*/*M*_an_, which considers the mean noise “*B*,” a known concentration of the studied analytes “*C*,” and the mean response of 20 samples spiked at a known concentration “*M*_an_” [8].

The Fc was determined by analyses of 20 blank samples spiked at the level of interest for each analyte, within the same day. This step was repeated again twice. The repetitions were carried out on three different days (*n* = 60). The analytical response was determined for the samples and for the two MRM transitions from each analytes, and the Fc was calculated using the following equation: Fc = *M*_an_ − 1.64 × SD_an_, which considers the mean response from the 60 samples “*M*_an_” and the standard deviation “SD” for each analyte (*n* = 60) and for two MRM transitions [8].

The detection capability (CC*β*) was evaluated by a comparison of the *T*-value and Fc. The *T*-value and Fc values obtained can lead to different situations: the first is when Fc > *T*-value, corresponding to the best situation, with a false-negative rate below 5%; in this case, the CC*β* is truly below the MRL level. However, when Fc < *T*-value, if the *T*-value is taken as a limit of positivity, more than 5% of the samples will be considered as negative. The consequence is that the CC*β* is truly above the MRL level [8].

The sensitivity of the method was determined by analyses of 20 blank samples, spiked at the level of interest, and compared with the Fc. The number of true positive samples giving a positive test result, also called “positive agreement”, was divided by the number of true positive samples and expressed as a percentage [8].

### 2.8. Applicability Demonstration by Animal Experiment Study

For the evaluation of applicability of the validated method, 600 Hy-Line W36 laying hens, with 40 weeks of age, were used. The birds were housed in production cages with ad libitum access to water and feed. The hens were randomly allocated into six experimental groups, labeled from A to F, containing 100 birds each. Hens from the A group formed the untreated control group and received nonmedicated feed throughout the experimental period, whereas those from groups B, C, D, E, and F received medicated feed containing neomycin, enrofloxacin, lincomycin, oxytetracycline, and doxycycline, respectively, during 5 days.

Before the initiation of treatment, 6 repetitions with a pool of 10 eggs each were collected from each group. Then, additional 6 repetitions of 10 eggs each were daily collected of all experimental groups, during the period of 15 days (5 days of treatment with medicated feed and 10 days of discontinuation of the medication). The samples were individually identified and sent to laboratory for UHPLC-MS/MS analyses using the previously validated method.

This study was carried out in strict accordance with the recommendations of the National Council for the Control of Animal Experimentation (CONCEA) at the Brazilian Ministry of Science and Technology and Innovation (MCTI). The protocol was approved by the Ethics Committee in Animal Experimentation at the* Universidade Federal de Minas Gerais* (UFMG) (Permit Number: 400/2015).

## 3. Results and Discussion

### 3.1. Mass Spectrometry Optimization

The operational conditions of the mass spectrometer were established by a direct infusion of the standards. The MRM transitions, monitored for each analyte, the declustering potential (DP), and the collision energy (CE) were optimized in order to improve the signal intensity. The relative ion intensity was evaluated according to the criteria established by European Commission Decision 2002/675/EC [6] and proved to be adequate for all the analytes (Tables 2 and 3).

### 3.2. Validation Study

**Figure 1:**
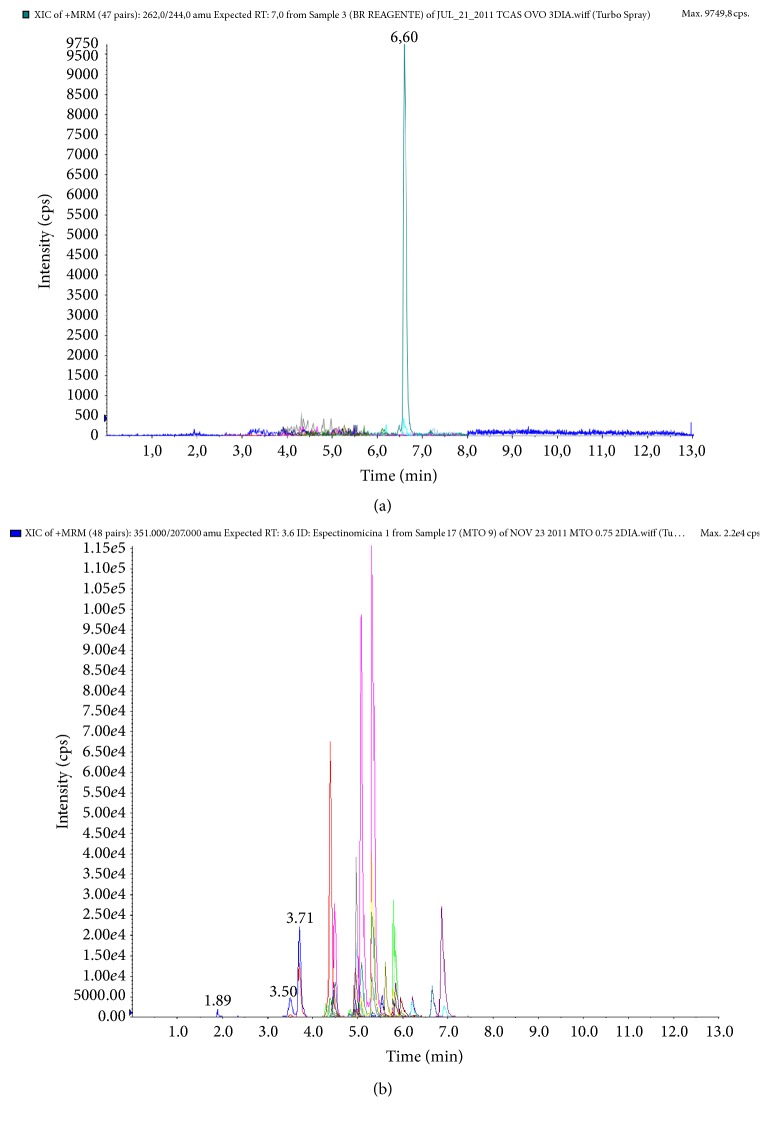
Chromatogram of a method blank-extraction with 5% trichloroacetic acid, without the addition of the egg matrix (a), to demonstrate the presence of an interferent compound from the TCA 5% solution and chromatogram of the egg matrix with the addition of the standard solutions of tetracyclines, aminoglycosides, quinolones, and lincosamides at the 0.75 MRL level (b).

According to the selectivity evaluation, the blank samples did not present interferences in the region of the studied analytes and signal suppression/enhancement by the egg matrix was not observed which compromised the detection of the analytes, except for the flumequine, which was eluted in the same retention time of the an interferent compound from the TCA 5% solution (Figure 1). This interferent compound provided a signal enhancement in the transition 262 > 244.

The detection capability (CC*β*) was evaluated by a comparison of the *T*-value and Fc. When Fc > *T*-value, the CC*β* is truly below the MRL level, indicating a false-negative rate below 5%. However, when Fc < *T*-value, if the *T*-value is taken as a limit of positivity, more than 5% of the samples will be considered as negative. The consequence is that the CC*β* is truly above the MRL level. The results obtained for the two transitions at validation concentration (*C*_val_) were satisfactory for all the studied analytes, except for the flumequine, where Fc was lower than the *T*-value in transition 1 and consequently the CC*β* was higher than the level of interest (Tables 4 and 5). This result may be justified due to the presence of an interferent compound that was eluted in the same retention time of this analyte.

The recommendation of the European Commission Decision 2002/675/EC [6] for screening methods is that they had the capability of a high sample throughput and allow the detection of the analytes of interest with a false-compliant rate below 5% at the level of interest; therefore the CC*β* of the method should be found below this level of interest. In the case of a suspected noncompliant result, this result should be confirmed by a confirmatory method.

The LOD of the method, for the analytes extracted with TCA, showed minimum values lower than 1 *μ*g kg^−1^ and the maximum values of 7.60 *μ*g kg^−1^. The highest LOD values were found for the analytes from the aminoglycosides family, followed by the antimicrobials from the tetracyclines family (Table 4). For the analytes extracted with acetonitrile, the minimum values obtained for the antimicrobials from the *β*-lactams family were lower than 1 *μ*g kg^−1^ with the maximum of 2.98 *μ*g kg^−1^, whereas, for the sulfonamides and macrolides families, the LOD was lower than 1 *μ*g kg^−1^ for all studied analytes (Table 5).

The sensitivity was 100% for the first transition of all analytes extracted by TCA, except for spectinomycin, which had a sensitivity of 95%. For the second transition, the sensitivity found was 100% for all analytes of these groups, with the exception of apramycin and dihydrostreptomycin, wherein the sensitivity was 95% (Table 4). For all the analytes extracted by acetonitrile, the sensitivity was 100% (Table 5). A sensitivity above 95% means that the CC*β* is below the level of concentration tested for validation; therefore the number of false-negatives is truly below 5% [8].

### 3.3. Applicability of the Method

The applicability of the method was evaluated by analyses of egg samples from hens that had been subjected to pharmacological treatment with the antimicrobials neomycin, enrofloxacin, lincomycin, oxytetracycline, and doxycycline administered via feed. Egg samples were classified as compliant or noncompliant by comparing their instrumental responses with the responses obtained in the analyzes of samples spiked at 0.75 times the validation concentration of each drug (Table 1), defined as positive control. Residues of neomycin, enrofloxacin and its metabolite ciprofloxacin, lincomycin, oxytetracycline, and doxycycline were not detected in any of the egg samples of hens from control group, indicating that there was no contamination of the feed and no cross-contamination during the treatments.

Residues of neomycin were not detected, during all the experimental period, in the egg samples from hens treated with neomycin. This drug has effective action in the gastrointestinal tract and is poorly absorbed from normal gastrointestinal tract and probably could not reach, in eggs, detectable concentrations by validated method.

**Figure 2:**
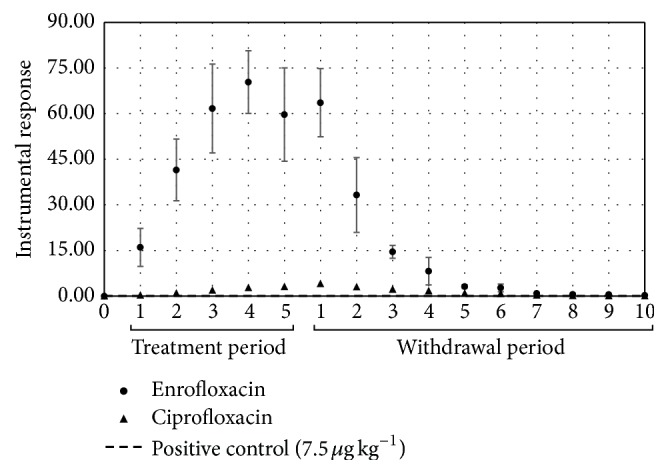
Residues of enrofloxacin and ciprofloxacin in egg samples from layer hens subjected to a pharmacological treatment with enrofloxacin, during the drugs administration period and days of residual evaluation.

In the egg samples from hens treated with enrofloxacin, a rapid increase in the levels of enrofloxacin residues was observed after initiation of drug administration. Residue concentrations of the drug that were higher than the reference limit (positive control) of 7.5 *μ*g kg^−1^ were found in the egg samples until nine days after the end of treatment. Ciprofloxacin showed similar characteristics; however, residue concentrations higher than the reference limit (positive control) were found up to six days after the discontinuation of the treatment (Figure 2); these results were expected, because ciprofloxacin is a metabolite of enrofloxacin.

The physiology of egg production is directly related to the accumulation of antimicrobial residues in eggs after laying. In the ovary of a laying hen, several follicles at varying developmental stages are present simultaneously. Before the laying of an egg, the yolk undergoes a phase of rapid growth, in which it increases in size exponentially over 10 days [25]. Hence, antimicrobials that deposit preferentially in the yolk will rapidly accumulate during this time and can be present in successive eggs for 10 or more days following treatment. Following maturation, the yolk moves to the magnum region of the oviduct and the majority of the albumen is deposited from secreted proteins, over a 2-3 h period, and may also serve as a residue accumulation site [2, 26]. Physicochemical properties of the drugs, such as its tendency to bind to plasma proteins, hydrophobicity, or hydrophilicity, also influence the distribution and persistence of residues in eggs and many drugs may deposit preferentially in the yolk or albumen according to those characteristics [27].

After oral administration, fluoroquinolones are rapidly absorbed, with high bioavailability [28–30], extensive metabolism, and distribution to tissues [29–31]. Although there is no general agreement regarding fluoroquinolone distribution, some authors described that these drugs accumulate mainly in the yolk [32, 33]. As the yolk has a long development time, which depends on a preovulatory hierarchy, residues of fluoroquinolones can be incorporated into this matrix, especially when the yolk undergoes a phase of rapid growth, in which it increases in size exponentially over 10 days due to incorporation of lipoproteins secreted in the liver [2, 25]. This may justify the long period of permanence of enrofloxacin residues in eggs after cessation of treatment, being detected in the egg samples, at concentrations higher than the reference limit (positive control) until nine days after the end of treatment.

**Figure 3:**
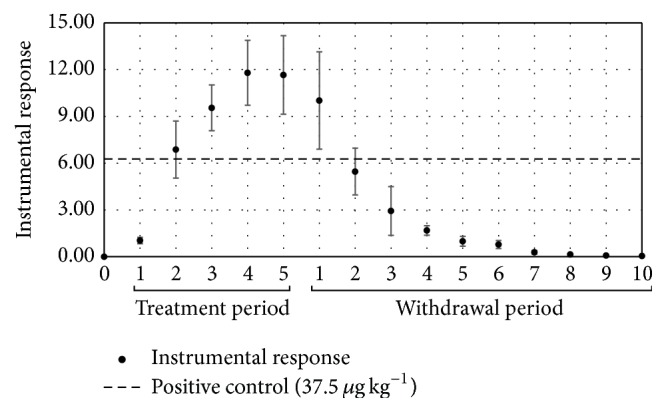
Residues of lincomycin in egg samples from layer hens subjected to a pharmacological treatment with lincomycin, during the drug administration period and days of residual evaluation.

In the group of hens treated with lincomycin, the highest concentrations of the drug were found during the treatment period. When the treatment was discontinued at the 5th day, the concentrations of lincomycin declined rapidly and were present in concentrations higher than the reference limit (positive control) of 37.5 *μ*g kg^−1^ up to one day after the discontinuation of the treatment (Figure 3).

Lincosamides are antimicrobials that have basic nature and high affinity for plasma proteins [34]. As the albumen is formed in the magnum region of the oviduct from secreted plasmatic proteins, generally formed on the previous day of posture [35, 36], the residue level of lincomycin probably rapidly reduces due to this property.

**Figure 4:**
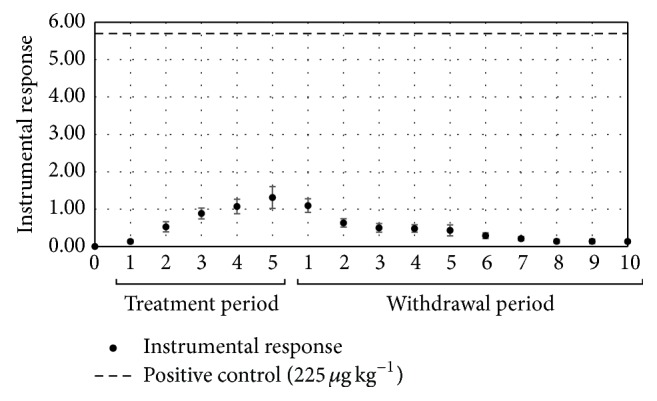
Residues of oxytetracycline in egg samples from layer hens subjected to a pharmacological treatment with oxytetracycline during the drug administration period and days of residual evaluation.

In contrast with the results observed for lincomycin, residues of oxytetracycline were below the reference limit (positive control) of 225 *μ*g kg^−1^ during all the experimental period (Figure 4).

Oxytetracycline is the least lipophilic member of the tetracycline group and consequently has a lower rate of absorption after oral administration, which may explain the observed results [34].

**Figure 5:**
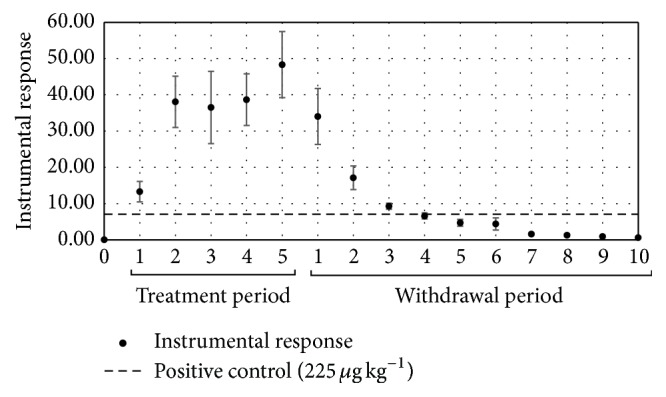
Residues of doxycycline in egg samples from layer hens subjected to a pharmacological treatment with doxycycline during the drug administration period and days of residual evaluation.

However, in the group of hens treated with doxycycline, residues of the drug were found at concentrations higher than the reference limit (positive control) of 225 *μ*g kg^−1^ up to four days after the discontinuation of the treatment (Figure 5).

Doxycycline is the most lipophilic of the tetracyclines and consequently has a high absorption and distribution [37]. According to literature data, after administration, residues of doxycycline increase rapidly and the concentrations found in egg white are much higher during treatment and 1 day after withdrawal. However, the levels in albumen decrease rapidly, whereas the concentrations reach higher levels and persist longer in egg yolk [38].

## 4. Conclusion

The results demonstrated the applicability of the proposed method that may be used in routine analysis as a qualitative or as a semiquantitative tool in order to identify the analyte present in the samples and direct it to analysis for a quantitative and confirmatory method, thereby reducing the costs of analyses. The evaluation of the eggs after antimicrobial administration to laying hens demonstrated the absence of neomycin, during all experimental period, and the presence of enrofloxacin, ciprofloxacin, lincomycin, and doxycycline at concentrations higher than the positive control level for each drug. Residues of oxytetracycline were found, but at low concentrations during all the experimental period.

## Figures and Tables

**Table 1:** Level of interest for individual analytes, according to the Codex Alimentarius^4^ and European Union^3^ MRL, for the method validation.

Analytes	MRL (*μ*g kg^−1^)	Validation concentration (*C*_val_) (*μ*g kg^−1^)
*Tetracyclines*		
Chlortetracycline + epichlortetracycline	400^a^	300
Doxycycline	Not established^a^	300^c^
Oxytetracycline + epioxytetracycline	400^a^	300
Tetracycline + epitetracycline	400^a^	300
*Aminoglycosides*		
Amikacin	Not established^b^	500^c^
Apramycin	Banned from being used in laying hens^b^	500^c^
Dihydrostreptomycin	No MRL in eggs^b^	500^c^
Gentamicin	No MRL in eggs^b^	500^c^
Hygromycin	Not established^b^	500^c^
Kanamicin	Banned from being used in laying hens^b^	500^c^
Neomycin	500^b^	500
Spectinomycin	Banned from being used in laying hens^b^	500^c^
Streptomycin	No MRL in eggs^b^	500^c^
Tobramycin	Not established^b^	500^c^
*Quinolones*		
Ciprofloxacin	Banned from being used in laying hens^b^	10
Enrofloxacin	Banned from being used in laying hens^b^	10
Flumequine	Banned from being used in laying hens^b^	10
Nalidixic acid	Not established^b^	10
Norfloxacin	Not established^b^	10
Oxolinic acid	Banned from being used in laying hens^b^	10
Sarafloxacin	Banned from being used in laying hens^b^	10
*Lincosamides*		
Lincomycin	50^b^	50
*β-Lactams*		
Cefazolin	No MRL in eggs^b^	50
Cloxacillin	Banned from being used in laying hens^b^	50
Dicloxacillin	Banned from being used in laying hens^b^	50
Nafcillin	No MRL in eggs^b^	50
Oxacillin	Banned from being used in laying hens^b^	50
Penicillin G	Banned from being used in laying hens^b^	50
Penicillin V	Not established^b^	50
*Sulfonamides*		
Sulfachloropyridazine	Banned from being used in laying hens^b^	10
Sulfadiazine	Banned from being used in laying hens^b^	10
Sulfadimethoxine	Banned from being used in laying hens^b^	10
Sulfadoxine	Banned from being used in laying hens^b^	10
Sulfamerazine	Banned from being used in laying hens^b^	10
Sulfamethazine	Banned from being used in laying hens^b^	10
Sulfamethoxazole	Banned from being used in laying hens^b^	10
Sulfamethoxypyridazine	Banned from being used in laying hens^b^	10
Sulfaquinoxaline	Banned from being used in laying hens^b^	10
Sulfathiazole	Banned from being used in laying hens^b^	10
Sulfisoxazole	Banned from being used in laying hens^b^	10
*Macrolides*		
Clindamycin	Not established^b^	150
Erythromycin	Not established^b^	150
Spiramycin	Banned from being used in laying hens^b^	150
Tilmicosin	Banned from being used in laying hens^b^	150
Tylosin	200^b^	200

^a^Codex Alimentarius Commission^4^; ^b^European Regulation number 37/2010^3^; ^c^for the analytes banned from being used in laying hens or those analytes that did not have an established MRL, the method was used only as a qualitative screening method.

**Table 2:** MRM transitions and MS/MS parameters for tetracyclines, aminoglycosides, quinolones, and lincosamides after optimization, in the trichloroacetic acid extract.

Analytes	DP^a^ (V)	Major transition (*m*/*z*)	CE^b^ (eV)	Minor transition 1 (*m/z*)	CE^b^ (eV)	Minor transition 2 (*m*/*z*)	CE^b^ (eV)	RT^c^ (min)	Relative intensity
*Tetracyclines*									
Chlortetracycline	61	479/98	67	479/275	55	—	—	6.0	84.4 ± 5.0
Doxycycline	55	445/428	25	445/154	40	—	—	6.3	5.8 ± 0.6
Oxytetracycline	41	461/201	59	461/283	53	—	—	4.9	50.1 ± 4.1
Tetracycline	55	445/410	27	445/427	25	—	—	5.2	25.9 ± 2.1
*Aminoglycosides*									
Amikacin	60	586/425	21	586/163	53	—	—	4.8	374.9 ± 52.7
Apramycin	82	540/217	35	540/378	25	—	—	5.2	112.6 ± 13.3
Dihydrostreptomycin	120	584/263	42	584/246	54	584/409	4	4.3	33.1 ± 3.2
Gentamicin	50	478/157	25	464/322	20	464/160	20	5.5	132.5 ± 20.1
Hygromycin	50	528/177	25	528/352	25	—	—	4.2	207.4 ± 19.9
Kanamycin	70	485/163	35	485/205	35	—	—	4.8	45.5 ± 5.7
Neomycin	120	615/161	41	615/296	35	—	—	5.9	25.9 ± 2.7
Spectinomycin	66	351/207	31	351/189	33	—	—	3.6	52.1 ± 2.6
Streptomycin	157	582/263	45	582/246	51	582/407	54	4.3	55.1 ± 7.4
Tobramycin	50	468/163	20	468/324	55	—	—	5.3	7.4 ± 2.0
*Quinolones*									
Ciprofloxacin	61	332/314	30	332/231	47	—	—	4.8	79.9 ± 12.1
Enrofloxacin	72	360/342	30	360/286	50	—	—	5.2	28.2 ± 5.2
Flumequine	44	262/244	25	262/202	45	—	—	7.0	9.6 ± 1.5
Nalidixic acid	42	233/215	30	233/187	35	—	—	6.8	96.2 ± 2.9
Norfloxacin	60	320/302	33	320/231	50	—	—	4.7	26.9 ± 4.2
Oxolinic acid	53	262/244	25	262/216	40	—	—	5.4	17.1 ± 1.6
Sarafloxacin	50	386/368	30	386/348	40	—	—	5.6	5.8 ± 2.2
*Lincosamides*									
Lincomycin	60	407/126	40	407/359	26	—	—	4.3	8.2 ± 0.7

^a^DP: declustering potential; ^b^CE: collision energy; ^c^RT: retention time.

**Table 3:** MRM transitions and MS/MS parameters for *β*-lactams, sulfonamides, and macrolides after optimization, in the acetonitrile acid extract.

Analytes	DP^a^ (V)	Major transition (*m/z*)	CE^b^ (eV)	Minor transition 1 (*m/z*)	CE^b^ (eV)	Minor transition 2 (*m/z*)	CE^b^ (eV)	RT^c^ (min)	Relative intensity
*β-lactams*									
Cefazolin	50	455/323	15	455/156	23	—	—	4.1	53.6 ± 3.3
Cloxacillin	50	436/160	20	436/277	20	—	—	8.3	120.5 ± 7.2
Dicloxacillin	50	470/160	20	470/311	20	—	—	8.6	65.9 ± 4.2
Nafcillin	50	415/199	20	415/171	50	—	—	8.4	31.4 ± 0.9
Oxacillin	50	402/160	18	402/243	18	—	—	8.0	105.4 ± 9.3
Penicillin G	70	335/176	21	335/160	21	335/114	37	7.3	103.7 ± 11.2
Penicillin V	66	351/160	15	351/192	17	—	—	7.7	9.9 ± 3.1
*Sulfonamides*									
Sulfachloropyridazine	51	285/156	21	285/92	39	—	—	4.6	39.4 ± 1.8
Sulfadiazine	53	251/156	22	251/108	30	—	—	2.6	39.2 ± 3.4
Sulfadimethoxine	50	311/156	23	311/108	37	—	—	6.1	30.8 ± 1.5
Sulfadoxine	60	311/156	25	311/108	40	—	—	5.0	30.8 ± 1.5
Sulfamerazine	60	265/92	35	265/156	35	—	—	3.1	44.6 ± 3.0
Sulfamethazine	50	279/156	25	279/108	36	—	—	3.6	76.8 ± 3.2
Sulfamethoxazole	60	254/108	35	254/92	35	—	—	5.0	117.3 ± 9.5
Sulfamethoxypyridazine	60	281/156	25	281/108	35	—	—	4.0	40.7 ± 2.0
Sulfaquinoxaline	50	301/156	23	301/108	40	—	—	6.2	33.6 ± 1.3
Sulfathiazole	53	256/156	20	256/108	34	—	—	3.1	33.6 ± 1.5
Sulfisoxazole	46	268/156	21	268/113	23	—	—	5.4	59.8 ± 5.0
*Macrolides*									
Clindamycin	75	425/126	43	425/377	27	—	—	6.9	8.9 ± 0.3
Erythromycin	66	734/158	43	734/576	27	—	—	7.6	50.3 ± 2.0
Spiramycin	56	422/174	31	422/101	25	—	—	7.4	65.7 ± 8.4
Tilmicosin	56	869/174	63	869/696	57	—	—	7.8	61.1 ± 3.4
Tylosin	115	916/174	55	916/772	43	—	—	7.8	35.1 ± 1.4

^a^DP: declustering potential; ^b^CE: collision energy; ^c^RT: retention time.

**Table 4:** Results of the method validation for tetracyclines, aminoglycosides, quinolones, and lincosamides families.

Analytes	Transition 1 “major”				Transition 2 “minor”			
	Fc^a^/*T*-value	CC*β*	LOD^b^ (*μ*g kg^−1^)	Sens^c^ (%)	Fc^a^/*T*-value	CC*β*	LOD^b^ (*μ*g kg^−1^)	Sens^c^ (%)
*Tetracyclines*								
Chlortetracycline	Fc > *T*	*<C*_val_^d^	<1	100	Fc > *T*	*<C*_val_^d^	<1	100
Doxycycline	Fc > *T*	*<C*_val_^d^	<1	100	Fc > *T*	*<C*_val_^d^	7.36	100
Oxytetracycline	Fc > *T*	*<C*_val_^d^	<1	100	Fc > *T*	*<C*_val_^d^	<1	100
Tetracycline	Fc > *T*	*<C*_val_^d^	<1	100	Fc > *T*	*<C*_val_^d^	1.08	100
*Aminoglycosides*								
Amikacin	Fc > *T*	*<C*_val_^d^	3.71	100	Fc > *T*	*<C*_val_^d^	3.29	100
Apramycin	Fc > *T*	*<C*_val_^d^	7.60	100	Fc > *T*	*<C*_val_^d^	5.22	95
Dihydrostreptomycin	Fc > *T*	*<C*_val_^d^	<1	100	Fc > *T*	*<C*_val_^d^	<1	95
Gentamicin	Fc > *T*	*<C*_val_^d^	4.68	100	Fc > *T*	*<C*_val_^d^	5.44	100
Hygromycin	Fc > *T*	*<C*_val_^d^	<1	100	Fc > *T*	*<C*_val_^d^	<1	100
Kanamycin	Fc > *T*	*<C*_val_^d^	<1	100	Fc > *T*	*<C*_val_^d^	4.87	100
Neomycin	Fc > *T*	*<C*_val_^d^	6.22	100	Fc > *T*	*<C*_val_^d^	6.91	100
Spectinomycin	Fc > *T*	*<C*_val_^d^	<1	95	Fc > *T*	*<C*_val_^d^	<1	100
Streptomycin	Fc > *T*	*<C*_val_^d^	<1	100	Fc > *T*	*<C*_val_^d^	<1	100
Tobramycin	Fc > *T*	*<C*_val_^d^	5.18	100	Fc > *T*	*<C*_val_^d^	7.01	100
*Quinolones*								
Ciprofloxacin	Fc > *T*	*<C*_val_^d^	<1	100	Fc > *T*	*<C*_val_^d^	<1	100
Enrofloxacin	Fc > *T*	*<C*_val_^d^	<1	100	Fc > *T*	*<C*_val_^d^	<1	100
*Flumequine*	*Fc <T*	*>C*_*val*_^*d*^	*<1*	*100*	Fc > *T*	*<C*_val_^d^	<1	100
Lincomycin	Fc > *T*	*<C*_val_^d^	<1	100	Fc > *T*	*<C*_val_^d^	<1	100
Nalidixic acid	Fc > *T*	*<C*_val_^d^	<1	100	Fc > *T*	*<C*_val_^d^	<1	100
Norfloxacin	Fc > *T*	*<C*_val_^d^	<1	100	Fc > *T*	*<C*_val_^d^	<1	100
Oxolinic acid	Fc > *T*	*<C*_val_^d^	<1	100	Fc > *T*	*<C*_val_^d^	<1	100
Sarafloxacin	Fc > *T*	*<C*_val_^d^	<1	100	Fc > *T*	*<C*_val_^d^	<1	100
*Lincosamides*								
Lincomycin	Fc > *T*	*<C*_val_^d^	<1	100	Fc > *T*	*<C*_val_^d^	<1	100

^a^Fc: cut-off factor; ^b^LOD: limit of detection; ^c^Sens: sensitivity of the method; ^d^*C*_val_: level of interest for each analyte according to Table 1.

**Table 5:** Results of the method validation for *β*-lactams, sulfonamides, and macrolides families.

Analytes	Transition 1 “major”				Transition 2 “minor”			
	Fc^a^/*T*-value	CC*β*	LOD^b^ (*μ*g kg^−1^)	Sens^c^ (%)	Fc^a^ /*T*-value	CC*β*	LOD^b^ (*μ*g kg^−1^)	Sens^c^ (%)
*β-lactams*								
Cefazolin	Fc > *T*	*<C*_val_^d^	<1	100	Fc > *T*	*<C*_val_^d^	2.98	100
Cloxacillin	Fc > *T*	*<C*_val_^d^	<1	100	Fc > *T*	*<C*_val_^d^	<1	100
Dicloxacillin	Fc > *T*	*<C*_val_^d^	<1	100	Fc > *T*	*<C*_val_^d^	2.10	100
Nafcillin	Fc > *T*	*<C*_val_^d^	<1	100	Fc > *T*	*<C*_val_^d^	2.35	100
Oxacillin	Fc > *T*	*<C*_val_^d^	<1	100	Fc > *T*	*<C*_val_^d^	<1	100
Penicillin G	Fc > *T*	*<C*_val_^d^	<1	100	Fc > *T*	*<C*_val_^d^	<1	100
Penicillin V	Fc > *T*	*<C*_val_^d^	<1	100	Fc > *T*	*<C*_val_^d^	1.76	100
*Sulfonamides*								
Sulfachloropyridazine	Fc > *T*	*<C*_val_^d^	<1	100	Fc > *T*	*<C*_val_^d^	<1	100
Sulfadiazine	Fc > *T*	*<C*_val_^d^	<1	100	Fc > *T*	*<C*_val_^d^	<1	100
Sulfadimethoxine	Fc > *T*	*<C*_val_^d^	<1	100	Fc > *T*	*<C*_val_^d^	<1	100
Sulfadoxine	Fc > *T*	*<C*_val_^d^	<1	100	Fc > *T*	*<C*_val_^d^	<1	100
Sulfamerazine	Fc > *T*	*<C*_val_^d^	<1	100	Fc > *T*	*<C*_val_^d^	<1	100
Sulfamethazine	Fc > *T*	*<C*_val_^d^	<1	100	Fc > *T*	*<C*_val_^d^	<1	100
Sulfamethoxazole	Fc > *T*	*<C*_val_^d^	<1	100	Fc > *T*	*<C*_val_^d^	<1	100
Sulfamethoxypyridazine	Fc > *T*	*<C*_val_^d^	<1	100	Fc > *T*	*<C*_val_^d^	<1	100
Sulfaquinoxaline	Fc > *T*	*<C*_val_^d^	<1	100	Fc > *T*	*<C*_val_^d^	<1	100
Sulfathiazole	Fc > *T*	*<C*_val_^d^	<1	100	Fc > *T*	*<C*_val_^d^	<1	100
Sulfisoxazole	Fc > *T*	*<C*_val_^d^	<1	100	Fc > *T*	*<C*_val_^d^	<1	100
*Macrolides*								
Clindamycin	Fc > *T*	*<C*_val_^d^	<1	100	Fc > *T*	*<C*_val_^d^	<1	100
Erythromycin	Fc > *T*	*<C*_val_^d^	<1	100	Fc > *T*	*<C*_val_^d^	<1	100
Spiramycin	Fc > *T*	*<C*_val_^d^	<1	100	Fc > *T*	*<C*_val_^d^	<1	100
Tilmicosin	Fc > *T*	*<C*_val_^d^	<1	100	Fc > *T*	*<C*_val_^d^	<1	100
Tylosin	Fc > *T*	*<C*_val_^d^	<1	100	Fc > *T*	*<C*_val_^d^	<1	100

^a^Fc: cut-off factor; ^b^LOD: limit of detection; ^c^Sens: sensitivity of the method; ^d^*C*_val_: level of interest for each analyte according to Table 1.
